# Virtual Fly Brain—An interactive atlas of the *Drosophila* nervous system

**DOI:** 10.3389/fphys.2023.1076533

**Published:** 2023-01-26

**Authors:** Robert Court, Marta Costa, Clare Pilgrim, Gillian Millburn, Alex Holmes, Alex McLachlan, Aoife Larkin, Nicolas Matentzoglu, Huseyin Kir, Helen Parkinson, Nicolas H. Brown, Cahir J. O’Kane, J. Douglas Armstrong, Gregory S. X. E. Jefferis, David Osumi-Sutherland

**Affiliations:** ^1^ School of Informatics, University of Edinburgh, Edinburgh, United Kingtom; ^2^ Department of Zoology, University of Cambridge, Cambridge, United Kingtom; ^3^ Department of Genetics, University of Cambridge, Cambridge, United Kingtom; ^4^ Department of Physiology, Development and Neuroscience, University of Cambridge, Cambridge, United Kingtom; ^5^ European Bioinformatics Institute (EMBL-EBI), Hinxton, United Kingtom; ^6^ MRC Laboratory for Molecular Biology, Cambridge, United Kingtom

**Keywords:** drosophila, atlas, connectomics, transcriptomics, neurobiology, ontology, FAIR

## Abstract

As a model organism, *Drosophila* is uniquely placed to contribute to our understanding of how brains control complex behavior. Not only does it have complex adaptive behaviors, but also a uniquely powerful genetic toolkit, increasingly complete dense connectomic maps of the central nervous system and a rapidly growing set of transcriptomic profiles of cell types. But this also poses a challenge: Given the massive amounts of available data, how are researchers to Find, Access, Integrate and Reuse (FAIR) relevant data in order to develop an integrated anatomical and molecular picture of circuits, inform hypothesis generation, and find reagents for experiments to test these hypotheses? The Virtual Fly Brain (virtualflybrain.org) web application & API provide a solution to this problem, using FAIR principles to integrate 3D images of neurons and brain regions, connectomics, transcriptomics and reagent expression data covering the whole CNS in both larva and adult. Users can search for neurons, neuroanatomy and reagents by name, location, or connectivity, *via* text search, clicking on 3D images, search-by-image, and queries by type (e.g., dopaminergic neuron) or properties (e.g., synaptic input in the antennal lobe). Returned results include cross-registered 3D images that can be explored in linked 2D and 3D browsers or downloaded under open licenses, and extensive descriptions of cell types and regions curated from the literature. These solutions are potentially extensible to cover similar atlasing and data integration challenges in vertebrates.

## 1 Introduction

Understanding the circuit basis of behavior is one of the grand challenges facing the biomedical sciences and has major implications for human society and health. Massive amounts of data that are relevant to this challenge are now available across multiple species. Dense connectomes covering a significant portion of the *Drosophila* central nervous system are available (Scheffer et al., 2020) and ongoing efforts are increasing coverage (Dorkenwald et al., 2020). Single-cell transcriptomic profiles, integrated with morphology and functional profiles, are available for a majority of cell types in the optic lobe (Kurmangaliyev et al., 2020; Özel et al., 2021) and more sparsely in other nervous system and brain regions (Davie et al., 2018; Li et al., 2022). In *Drosophila*, transgenic techniques (Luan et al., 2006; Pfeiffer et al., 2010) and libraries of transgenes (Jenett et al., 2012; Tirian and Dickson, 2017) allow precise targeting of neuron types to manipulate and measure their activity, enabling the genetic dissection of circuit function.

While this opens up unprecedented opportunities for understanding the circuit basis of behavior, it also poses new challenges: How can researchers know when they have data about one of the thousands of new cell types being identified and characterized in the literature and as part of large-scale analyses? How can they explore these massive new datasets, in conjunction with the literature, to generate hypotheses and form an integrated picture of the anatomical and molecular nature of circuits? How can researchers share their data in a way that conforms to FAIR standards (Wilkinson et al., 2016) and easily reuse the data of others?

Virtual Fly Brain (VFB) (Milyaev et al., 2012) provides a solution to these problems by integrating massive amounts of data derived from diverse techniques and multiple sources along with curated information from the literature. All this content is available *via* a web application and an API. The web application facilitates finding and integrating information about brain regions, neuron types and individual registered images, *via* sophisticated text search, point and click interaction with 3D images and graph visualizations and *via* semantic queries (e.g., finding neurons by type and location). All data is integrated following FAIR principles and we provide tools to enable users to share and integrate their data on VFB following these principles. VFB integrates images, connectomics and transcriptomics data by using two strategies - semantic and image-based.

Semantic integration is facilitated by the *Drosophila* Anatomy Ontology (DAO) (Costa et al., 2013), curated from the literature, and increasingly from data-driven identification of cell types. The DAO serves both as a queryable store of knowledge about *Drosophila* neuroanatomy, cell types and their classifications, and a source of terms for annotating data across modalities including images, connectomics, transcriptomics and expression patterns. Standard parcellation schemes have been developed for all *Drosophila* central nervous system regions and stages including adult brain and ventral nerve cord (Ito et al., 2014; Court et al., 2020). These parcellation schemes provide a standard reference for CNS regions defined in the DAO. Following FAIR data standards, all data on VFB is accessible *via* an identifier, in the form of a URL, that is globally unique, persistent, and resolvable.

**FIGURE 1:**
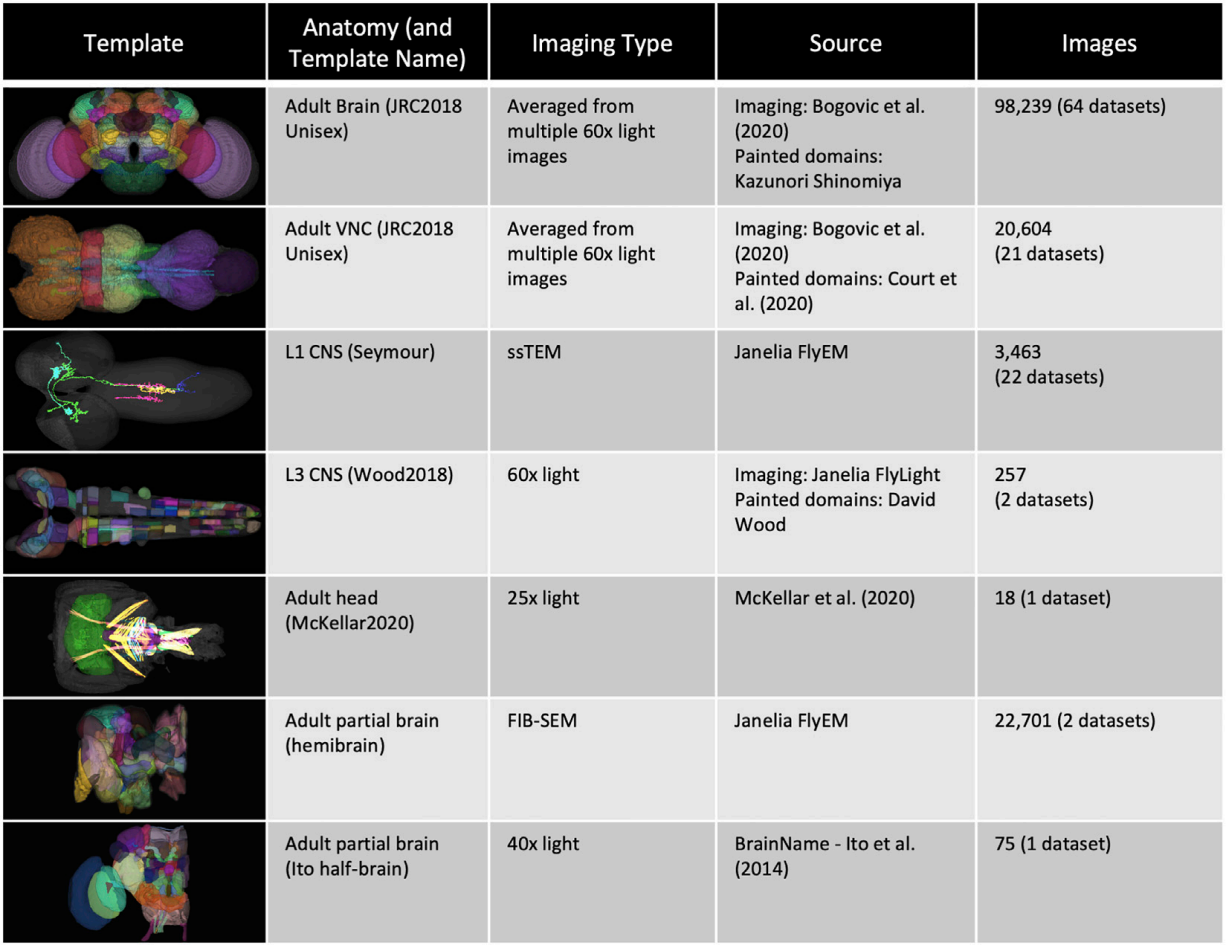
*Templates and content*. VFB has templates that integrate image data into common coordinate spaces and many more that serve as references for datasets in their native space The first four rows of the table provide details of the most up-to-date integrative templates on VFB: the JRC2018 unisex adult brain template (Bogovic et al., 2019) has the largest number of aligned images, which include over 26,500 EM images from CATMAID FAFB (Zheng et al., 2018) and the Janelia Hemibrain (Scheffer et al., 2020) combined, over 70,000 images showing expression patterns or fragments of expression patterns from sources including FlyCircuit (Chiang et al., 2010; Shih et al., 2015) and Janelia FlyLight (Meissner et al., 2022) and 46 painted neuropil domains (painted by Kazunori Shinomiya); The JRC2018 adult ventral nerve cord (VNC) template (Bogovic et al., 2019) has over 2,000 EM images from CATMAID FANC (Phelps et al., 2021), over 18,000 images of expression patterns from sources including FlyLight and 21 painted domains (Court et al., 2020) The Seymour L1 template has nearly 3,500 EM images from CATMAID L1 (Ohyama et al., 2015); The Wood2018 template has 255 painted domains (David Wood and Volker Hartenstein, unpublished). The rest of the table provides details of three of the available reference templated on VFB: the McKellar2020 adult head template has painted domains showing the adult pharyngeal musculature (McKellar et al., 2020); the hemibrain has the hemibrain connectome in its native space, along with a more detailed parcellation scheme (Scheffer et al., 2020); the Ito half-brain is the original reference template and parcellation scheme for the BrainName standard (Ito et al., 2014).

Image-based integration makes use of standard templates (see Figure 1) onto which image data is registered (morphed), allowing hundreds of thousands of images from multiple imaging modalities to be co-registered so that they can be viewed and analyzed programmatically in a common coordinate space. Cross-registration has made it possible to design search algorithms to compare neurons, including NBLAST (Costa et al., 2016), which provides a similarity score for any two cross-registered neuron tracings based on how similar their morphology and location are. These and other alignment-style queries are key to solving another problem—that of defining neuron types in ways that allow them to be identified from data using quantitative criteria rather than, as traditionally, using qualitative criteria and human judgment. A similar problem was solved in genomics by the use of BLAST in combination with versioned genome builds, annotated with gene models. Registered 3D neuron images can be mapped to a type using NBLAST, as long as we have a set of reference images for neuron types. While the concept of a gene is hard to define non-controversially (Portin and Wilkins, 2017), and gene model annotation can be error prone and controversial (Koonin and Galperin, 2003), there is enough shared understanding and agreement to use sequence similarity to map genomic and transcriptomic sequence data to specific genes. The concept of cell type is even more controversial (Bates et al., 2019; Zeng, 2022), but neurobiologists typically group cells sharing morphological, connectomic, functional and developmental properties together under a common name, and generally refer to such groupings as types. For neurons in the *Drosophila* nervous system, shared location and morphology is highly indicative of shared developmental origin, connectivity and response properties (Bates et al., 2019). Neurons with shared location and morphology can be consistently identified across individuals and are present in numbers from 1–1000 per brain hemisphere (Bates et al., 2019). Shared morphology is therefore a strong indicator of cell type.

Clustering neurons with similar morphology based on NBLAST score identifies many previously identified types (Costa et al., 2016) indicating that NBLAST can be used reliably in many cases to identify neuronal type. In the case of sequence data, annotated reference genomes provide a reference standard for gene identity. While we have no equivalent standard reference for cell type morphologies, the availability of large connectomics projects with annotated neuron types, assessed at least in part using morphology *via* NBLAST scores, has provided us with a good *de facto* standard. For example, the largest of these published to date (Scheffer et al., 2020) is represented on VFB along with mappings to >1,100 known types and assigned a further 3864 provisional types based on NBLAST similarity—all cataloged and classified using DAO neuron type terms. VFB can therefore support cell type identification from data using NBLAST for a large and growing set of neuron types, assigning standard aoverlapping functionality such as NeuronBridge (Clements et al., 2022) and neuprint (Clements et al., 2020), are to support data-discovery across many sources, to make first-pass exploration of complex data easy and to link that data to the literature. For further analysis, users can download data, or follow links from data on VFB to the same data on these other resources with their own data downloads, data exploration and search tools.

We believe that this initial data discovery and exploration step will become increasingly important as more and more single-cell transcriptomics and connectomics datasets become available. Consistent semantic annotation across diverse datasets on VFB is key to achieving this. Not only does this support matching of cell types across datasets, it also allows for sophisticated queries that group data in biologically relevant ways, for example by gross classification, location or connectivity of annotated neurons.

## 2 Methods

### 2.1 Curation

VFB curators work closely with FlyBase, the EBI single cell expression atlas curators and data providers to curate information from the literature and annotate data in a timely manner. Literature curation captures information about neuron types and transgene expression and takes advantage of FlyBase curation, including community curation efforts and text-mining pipelines (Bunt et al., 2012; Halperin et al., 2012; Larkin et al., 2021) to easily identify and prioritize papers that contain data of high priority for VFB curation. Data curation standardizes the annotation of neuron types and transgenes in data using the same ontology and feature identifiers as FlyBase.

### 2.2 Semantic integration

#### 2.2.1 Ontologies and semantic schemas

VFB is built around the *Drosophila* Anatomy Ontology (DAO) (Costa et al., 2013), a manually curated, query-able classification of *Drosophila* anatomical structures and cell types expressed in Web Ontology Language (OWL) (World Wide Web Consortium, 2012). DAO is built using community standards (Jackson et al., 2021) and tooling (Matentzoglu et al., 2022) for sustainable, scalable ontology development. Neuroanatomy is represented in DAO using a standard schema that supports recording neuronal location, connectivity, lineage and function and incorporates basic spatial reasoning (Osumi-Sutherland et al., 2012). We have extended this schema to incorporate relations for recording brain regions in which a neuron type has its *major* inputs and outputs, for example, that the synaptic input onto DA1 uniglomerular antennal lobe projection neurons is concentrated in the DA1 glomerulus (Stocker et al., 1990; Bates et al., 2020a) distinguishing these from small numbers of inputs and outputs that occur on almost all parts of any neuron in connectomics data. Using this schema and information curated from over 1000 papers, the DAO represents 13,000 neuroanatomical structures and cell types, including over 9800 terms for neuron types (e.g. DL1 adPN) and more general neuron classifications (e.g. “cholinergic neuron”, “uniglomerular antennal lobe projection neuron”). The neuron types include over 3800 that are predicted from connectomics data (Scheffer et al., 2020) and over 2750 types for which we have curated lineage, which is reflected in links to neuroblast (e.g. develops from BAl3p neuroblast), lineage clones (e.g. part of BAl3p lineage clone) and classifications (e.g. BAl3p lineage neuron). This ontology and OWL schema, along with an OWL schema for representing image metadata and image registration (Osumi-Sutherland et al., 2014), are also used to classify and record the properties of cell types depicted in 3D images, connectomics and transcriptomics data on VFB. This means that the same OWL queries can be used both to query for data about individual neurons, and also to drive searches for neuron types based on their classification and properties (see, for example, the compound query in Figure 4).

**FIGURE 2:**
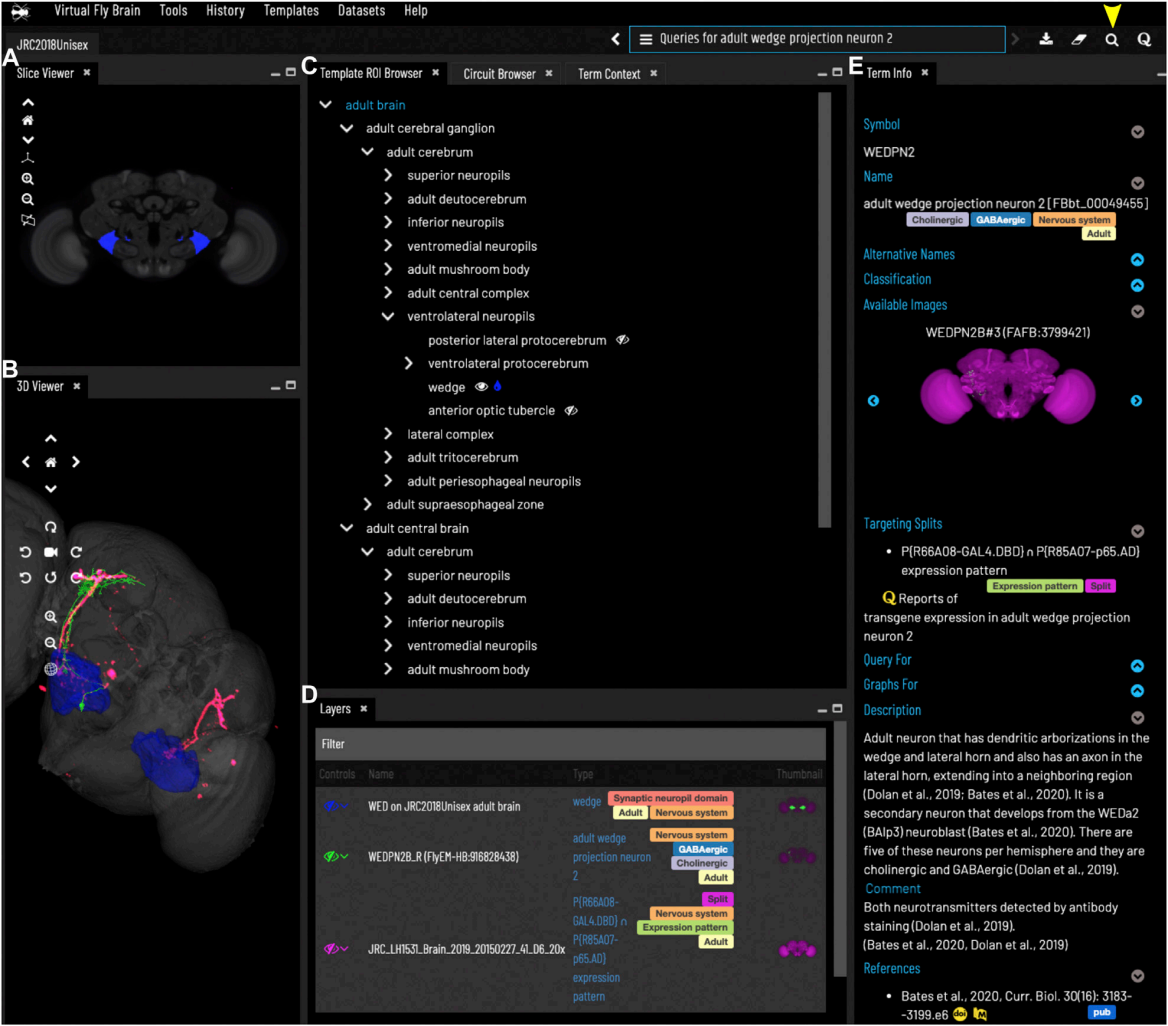
*General layout and browsing*. **(A)** The Slice Viewer allows users to view single slices of the Z-stack of the displayed elements. **(B)** The 3D Viewer shows entities in 3D space, allowing zoom and rotation. **(C)** The Template ROI Browser shows the neuropil regions of the current template (arranged hierarchically) and allows these to be added to the display. **(D)** The Layers tool acts as a color key for all the entities currently loaded and features a set of controls allowing content to be removed, hidden or recolored. **(E)** The term info shows details of a selected entity, in this case a cell type. Available images of this cell type are shown as thumbnails and can be added to the viewer by clicking the thumbnail. Split-GAL4 lines that target this cell type are also shown in the Term Info. Cell types also have a description based on published information. Term Info for a different entity can be shown by clicking on something in the Term Info or Layers panes, searching or using the left/right arrows above the Term Info pane. Arrowhead at top right indicates the search tool.

**FIGURE 3:**
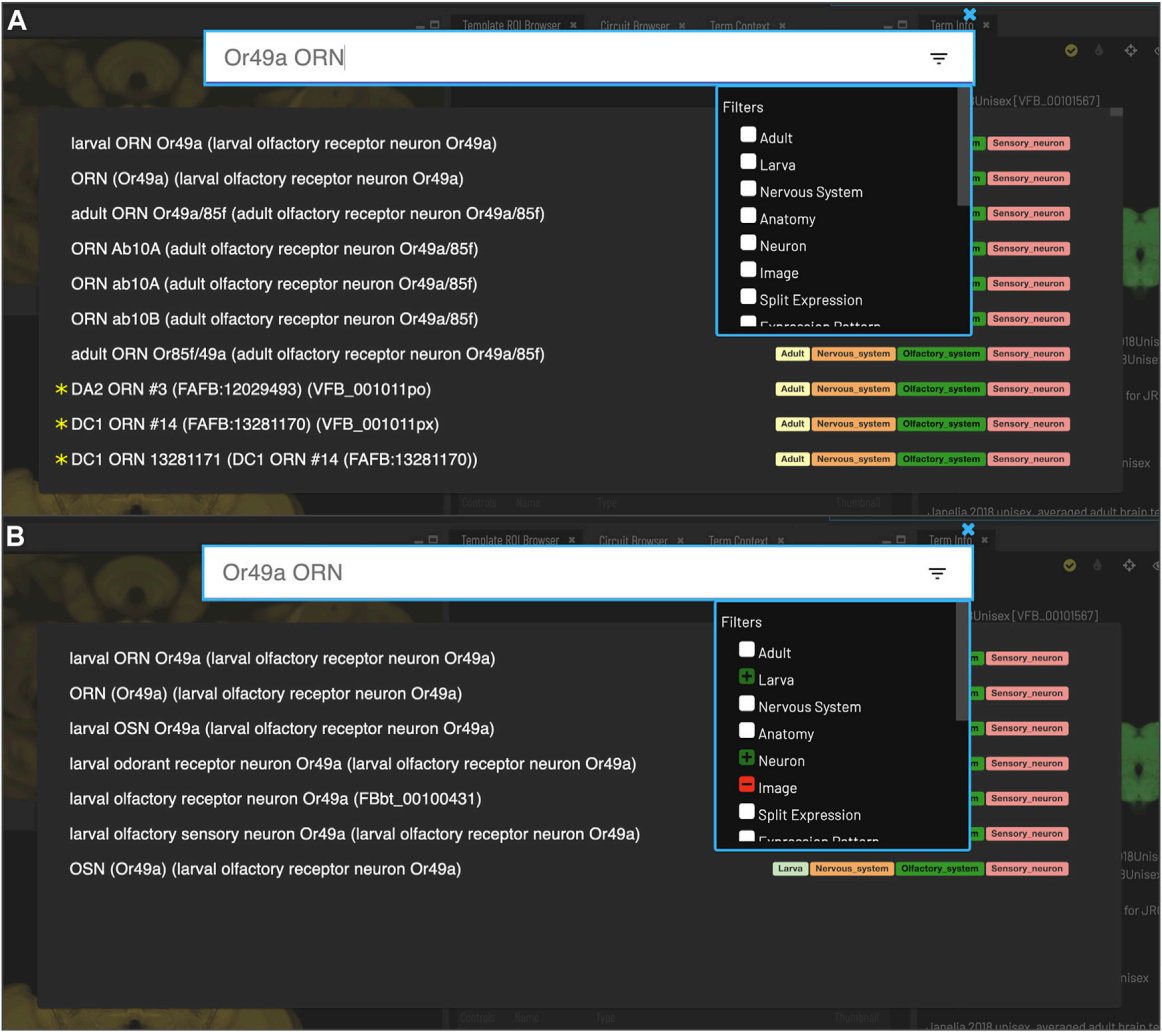
*Search*. Clicking the magnifying glass in the top right of the page will open the Search tool. Searching based on synonyms is supported and semantic tags on the right of each result provide extra information. Filters can be accessed by clicking on the lines on the right. **(A)** With no filters applied, results for “Or49a ORN” are a mixture of images (marked with *) and cell types from adult and larval stages. **(B)** To restrict results to larval neuron types, excluding images, filters can be applied to narrow down the results list, choosing a positive filter (green) for Larva and Neuron, and a negative filter (red) for Image.

**FIGURE 4:**
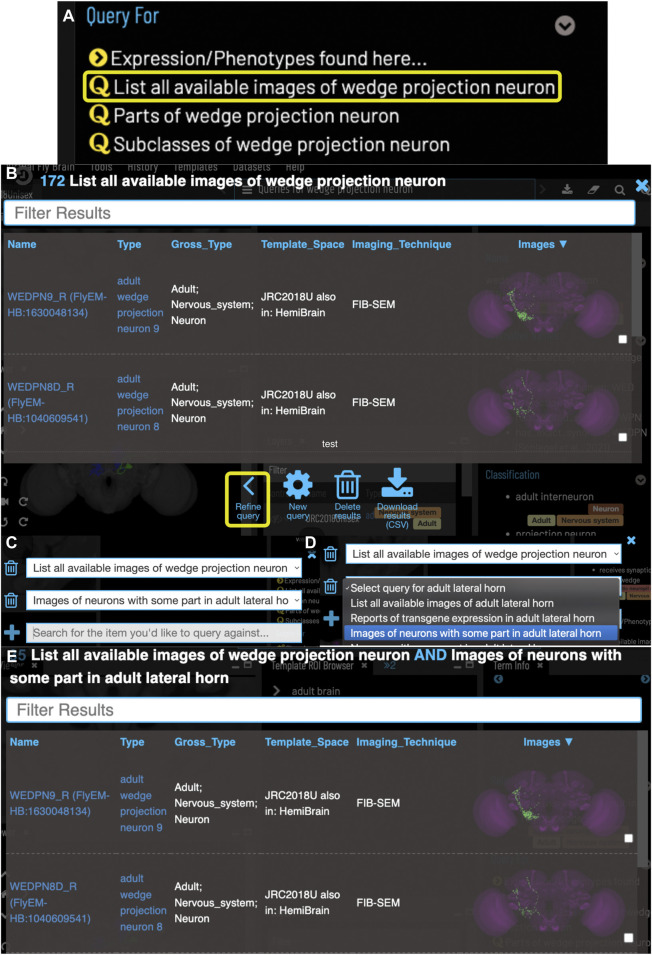
Compound Queries. The Term Info pane **(A)** shows queries available in the TermInfo of “wedge projection neuron”. Clicking on the query for available images of “wedge projection neuron” bring up a results table **(B)** which can be further refined by clicking “Refine Query” underneath. The query interface **(C,D)** shows the original query and allows a second query to be run to find items that fit both sets of criteria, in this case images of neurons that also have some part in the lateral horn. Images in the subsequent results table **(E)** can be added to the viewer by clicking the checkboxes on the right.

**FIGURE 5:**
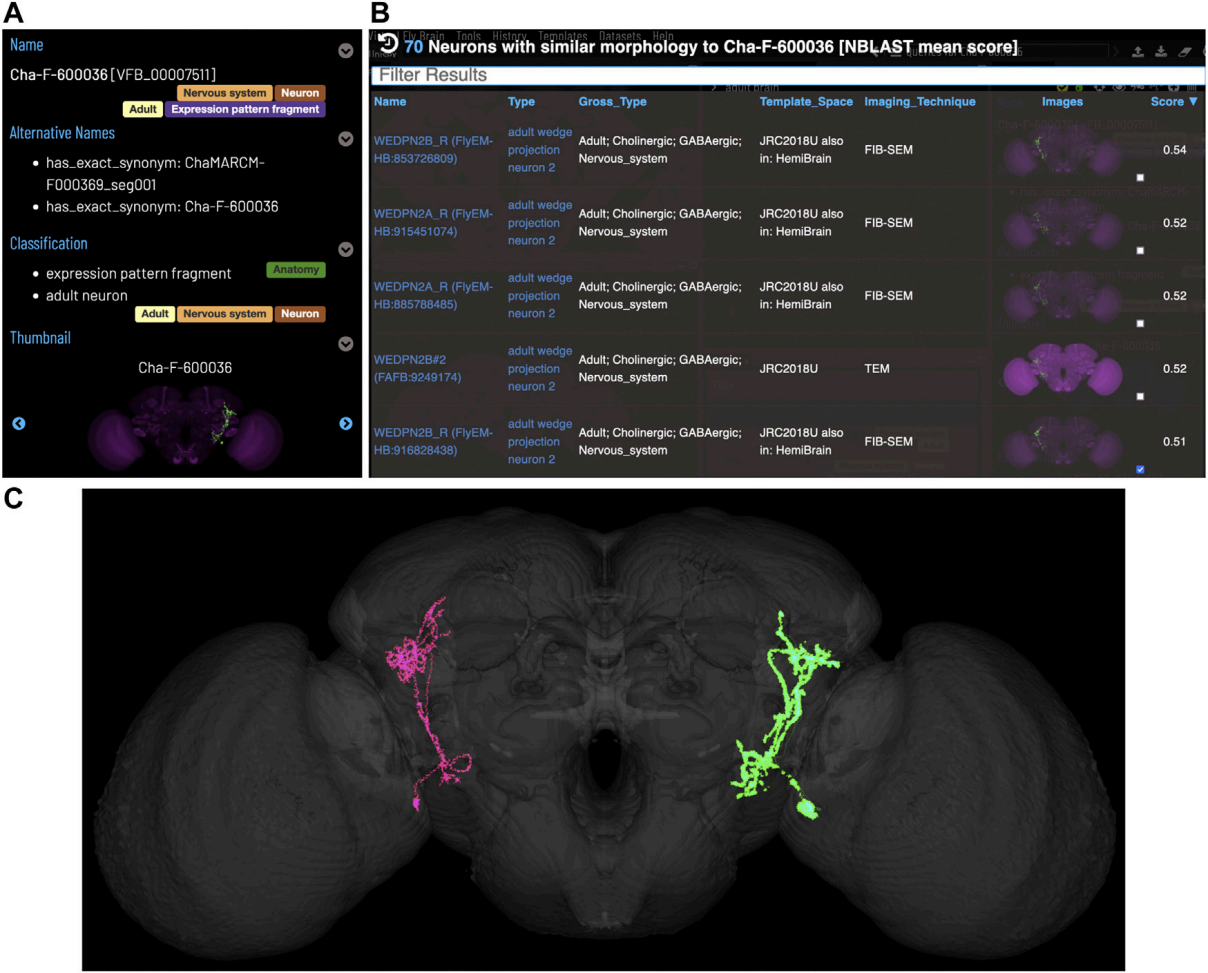
*Typing neurons using NBLAST*. **(A)** TermIinfo for a neuron from FlyCircuit (Chiang et al., 2010) with no curated type other than “neuron”. **(B)** NBLAST query results for neurons similar morphology to the untyped query neuron. The top five results are all typed as “adult wedge projection neuron 2”. **(C)** image comparing the morphology of the query FlyCircuit neuron [“Cha-F-600036 (VFB_00007511)” in green] and the ‘adult wedge projection neuron 2' “WEDPN2B_R (FlyEM-HB:916828438) [VFB_jrchk7yi]" WEDPN2B_R in magenta).

**FIGURE 6:**
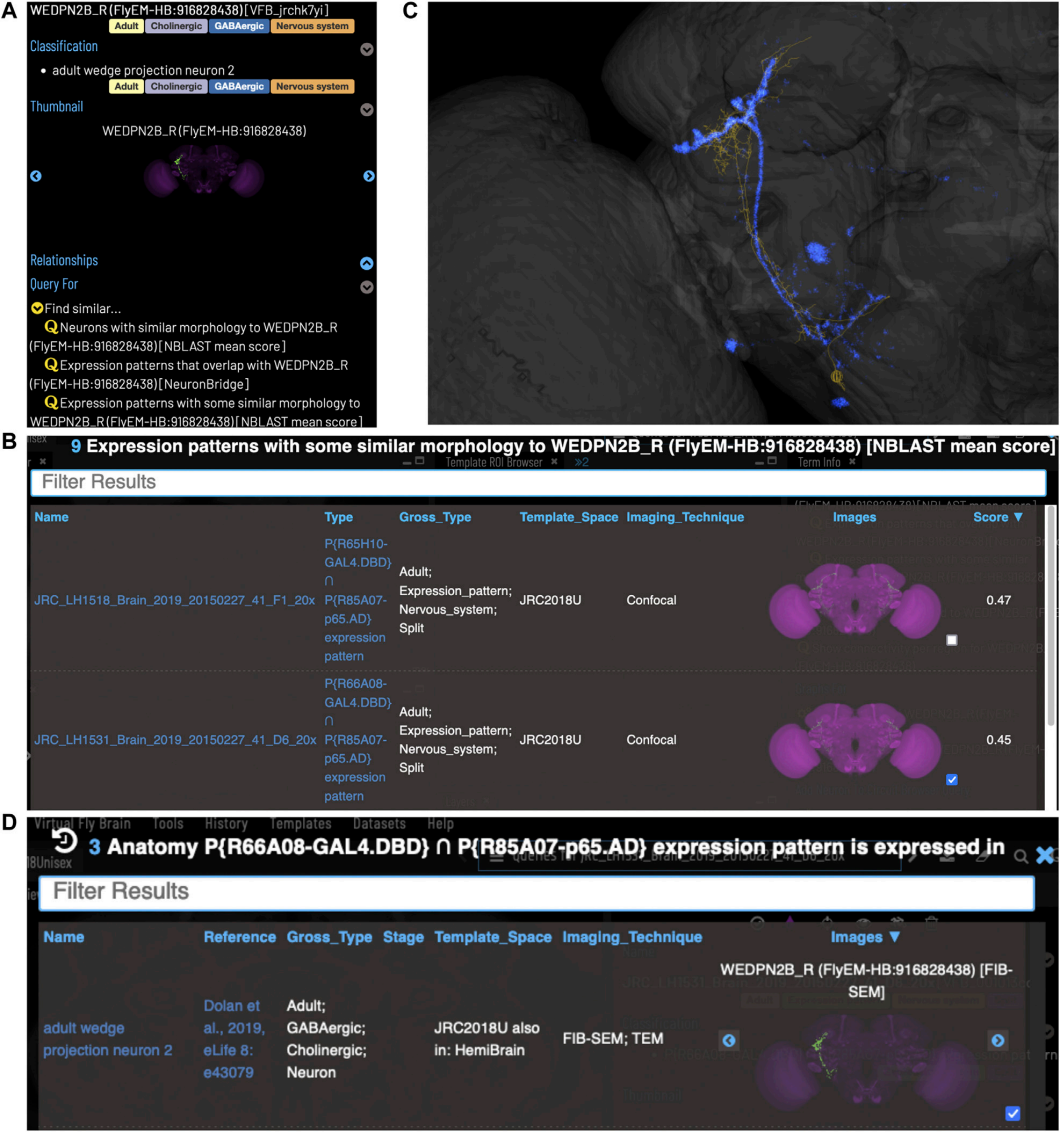
Identifying Split-GAL4 combinations that potentially target a query neuron. **(A)** TermInfo for a neuron, “WEDPN2B_R”, type “adult wedge projection neuron 2” (WEDPN2), from the hemibrain dataset. This neuron will be used for an NBLAST search. **(B)** NBLAST query results showing Split-GAL4 driver line results and NBLAST scores. The third result (with the checked tickbox) was selected for further investigation (panels C and D). **(C)** Image of the query neuron skeleton (yellow) and Split-GAL4 expression pattern point cloud (blue) overlap. **(D)** A search for neuron types that this Split-GAL4 combination is known to target, curated from the literature, finds the type of the neuron used for the NBLAST search (WEDPN2), supporting the NBLAST query result in this case.

**FIGURE 7:**
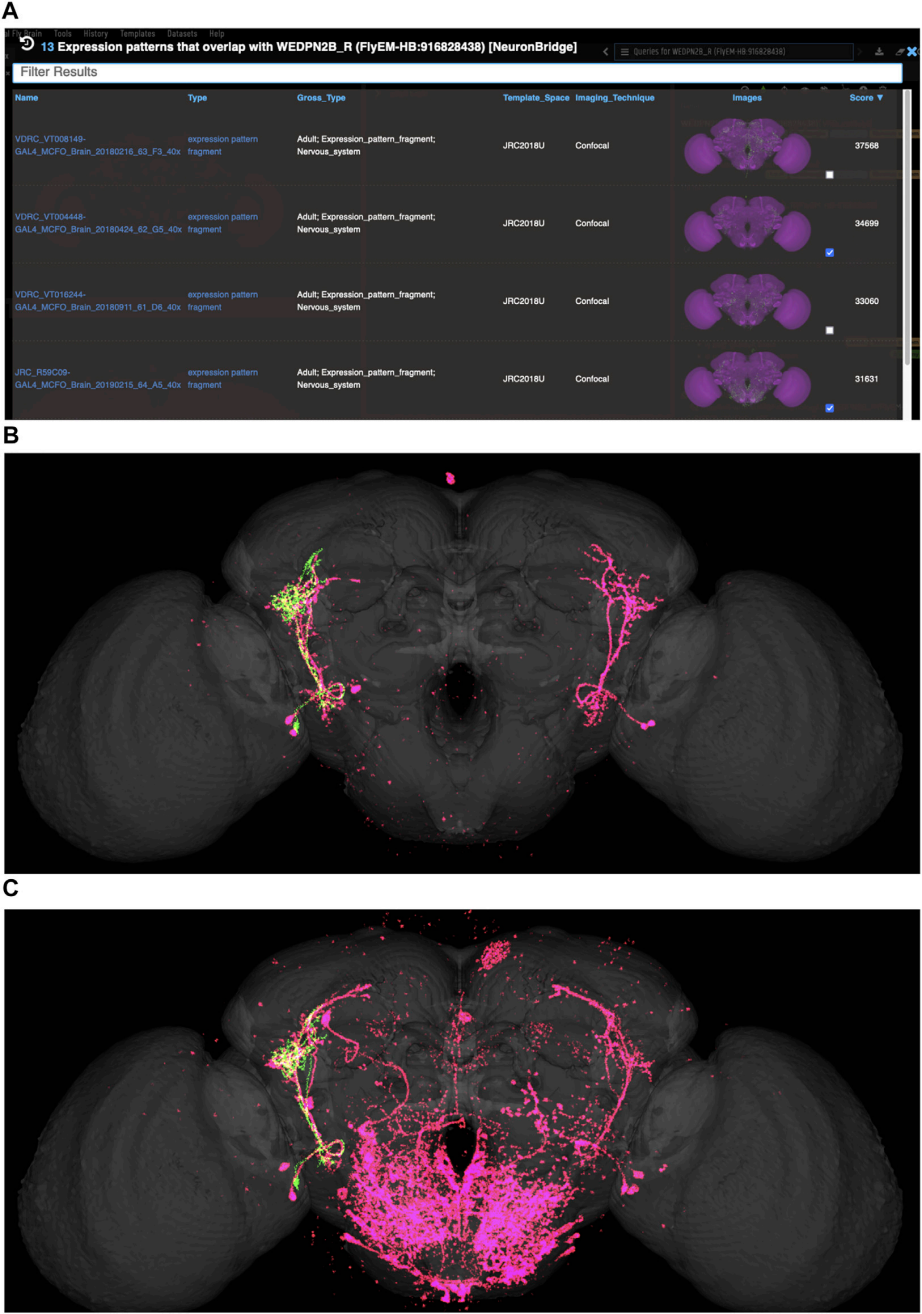
Identifying potential GAL4 drivers using color depth MIP scores **(A)** query results for the neuron “WEDPN2B_R”, showing hits to multiple MCFO images of driver line results and color depth MIP scores. The second (sparse MCFO expression) and fourth (dense MCFO expression) results (checked tickboxes) were selected for further investigation (panels (B) and (C), respectively). **(B)** Image of the query neuron (green) and expression pattern point cloud from a sparse line (magenta) overlap. **(C)** The same query neuron (green) also overlaps with the expression pattern point cloud of a dense line (magenta).

**FIGURE 8:**
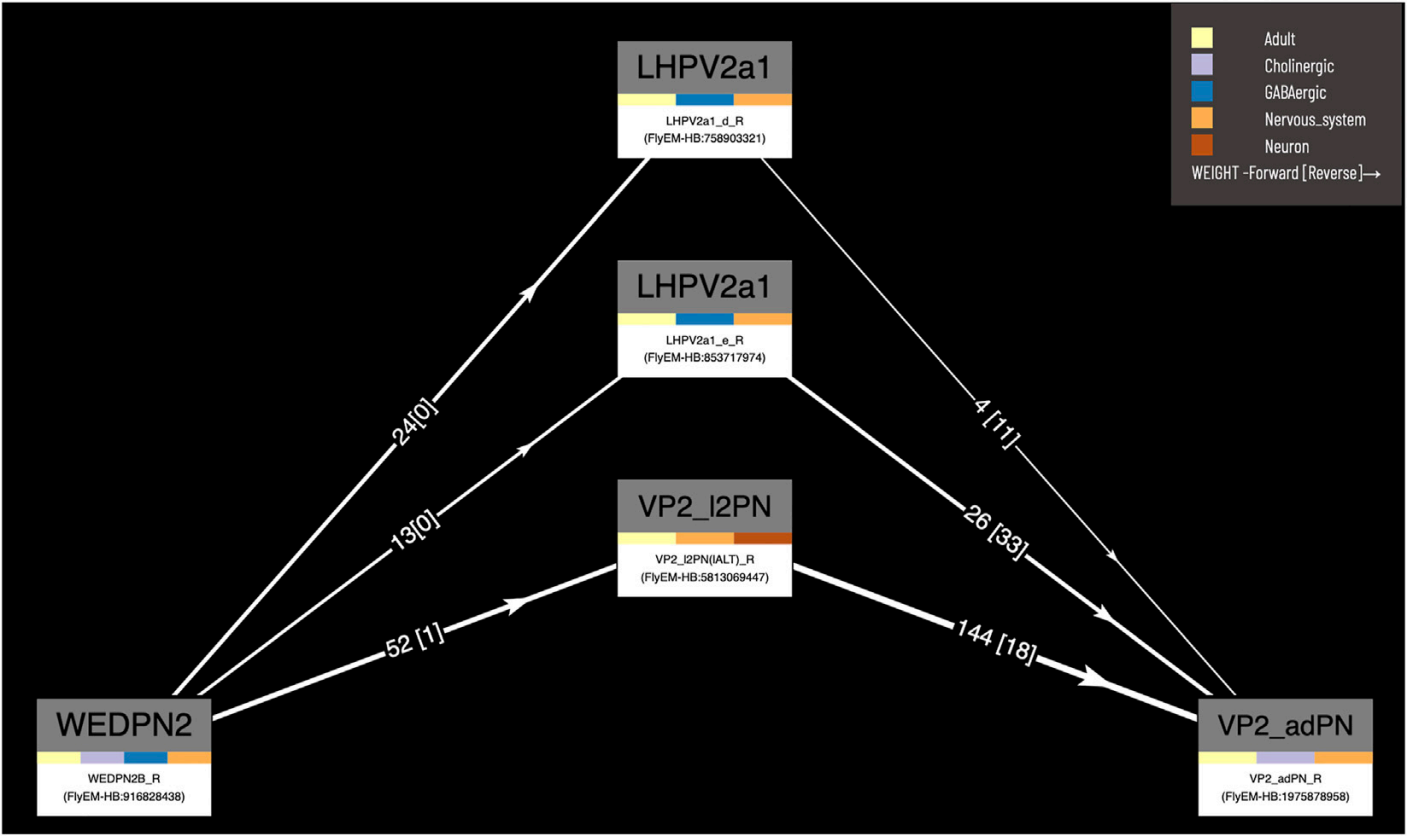
*Circuit browser.* A circuit diagram of paths between “WEDPN2B_R” and “VP2_adPN_R”. Rectangles represent neurons with the symbols of classes at the top, names of individual neurons at the bottom and colors corresponding to gross classifications in the middle. The legend for these gross classifications can be seen in the top-right [note the WDPN2 is classed as both cholinergic and glutamatergic based on antibody staining evidence (Dolan et al., 2019)]. Pathways are ordered from “strongest” at the bottom to “weakest” at the top. Arrows show the direction of synaptic connectivity and numbers outside of brackets show the number of synapses annotated for each connection. Numbers inside brackets show the number of synapses in the opposite direction.

The common OWL schema is also used to drive a system of semantic tags - short, informative pieces of text (e.g., cholinergic, larval, synaptic_neuropil) that appear as badges attached to ontology terms and data on the VFB site (Figures 2, 4–8) and are used to drive filters for text search (Figure 3).

The DAO is also used to annotate transgene expression patterns and single cell RNAseq data in FlyBase—the latter as part of a collaboration between FlyBase and the EBI single cell expression atlas. We convert all of this into a standard OWL representation for import into VFB. The spatial reasoning built into the DAO allows us to provide a highly enriched set of results when users query for transgenes expressed in an anatomical structure—returning transgenes expressed in neurons that have some part in this structure or any of its substructures (Milyaev et al., 2012; Osumi-Sutherland et al., 2012).

While OWL has many advantages for standardization and querying, it cannot serve all VFB use cases. OWL is not designed for fast, tunable text search with autosuggestion. For this VFB uses an Apache SOLR document store. It is also not ideally suited for automatically generating graph and tree visualizations or for maintaining and updating image annotations. The graph database Neo4J (Neo4j Graph Data Platform, 2020) is ideal for both of these use cases and provides a parallel system for graph pattern queries (e.g. for image metadata) that is simpler and more flexible than SPARQL (McCarthy et al., 2012), the standard graph-pattern query language for OWL. We developed a standard translation between OWL and Neo4j, covering a limited subset of OWL, optimized for readability and queryability and supported by a Java Library (Tan et al., 2021). This allows us to maintain a curation database in Neo4J (VFB-KB) and a front-facing Neo4j server for generating trees (Figure 2C), graphs (Figure 8) and graph queries for the VFB web-app and API.

#### 2.2.2 Data integration pipeline and servers

The VFB extract transform and load (ETL) pipeline extracts data from diverse sources (FlyBase (Gramates et al., 2022), CATMAID (Saalfeld et al., 2009), NeuronBridge (Clements et al., 2022), NeuPrint (Clements et al., 2020)) into ROBOT templates (Jackson et al., 2019) specifying their transformation to OWL following our standard schemas. We then load the resulting OWL files into a triple store, along with the various ontologies used in data annotation (also in OWL) and an OWL version of our curation database (VFB-KB). The triple store integrates all of this content around a common set of persistent URLs that serve as identifiers for ontology terms, data instances etc, merging and deduplicating references to these entities. Downstream of the triplestore, a pipeline adds semantic tags using OWL and SPARQL queries, and loads the front-facing servers.

### 2.3 Image integration and NBLAST

Unregistered images were registered using CMTK with nine degrees of freedom followed by a non-rigid registration (Rohlfing and Maurer, 2003; Jefferis et al., 2007). If necessary, data was moved to the left side of the brain by flipping and then applying a mirroring registration (Bates et al., 2020b). We made use of standard bridging registrations wherever possible to cross-register images from external templates, or between templates on VFB. For images registered to templates not hosted on VFB or where we needed to move between templates on VFB, we made use of bridging registrations wherever possible (Bates et al., 2020b).

The NBLAST implementation in Navis (Bates et al., 2020a) was used to generate a complete NBLAST matrix comparing all single neuron skeletons in VFB, including skeletons from the Janelia hemibrain (Scheffer et al., 2020), FAFB (Zheng et al., 2018) and FlyCircuit (Chiang et al., 2010) datasets) with each other and with all split-GAL4 expression patterns in the VFB database registered to the JRC2018 adult unisex brain template (Bogovic et al., 2019). Most neuron types are present as bilaterally symmetric pairs. To match the same type on opposite sides of the brain, each neuron-to-neuron NBLAST was performed and then repeated with one of the neurons mirrored along the midline and only the highest of these two scores was retained. Split-GAL4 expression patterns almost always label the same neuron(s) on both sides of the brain, so we used NBLAST to compare a union of each neuron and its mirror image across the midline to each split-GAL4 expression pattern. For NBLAST between neurons and from neurons to split-Gal4 expression patterns, mean scores were calculated so that a single score represents each pairwise comparison, regardless of direction. This biases the results towards sparse expression patterns, minimizing off-target expression and avoids promoting high scoring matches from neuron fragments to whole neurons, as the score for whole neuron to fragment in these cases will be low. In cases where a neuron is known to be truncated at the edge of a sample, the neuron being compared to it is also truncated to the same boundary before the mean NBLAST score is calculated. Queries on VFB (Figures 5–7) use precomputed NBLAST scores for neuron to neuron and neuron to Split-Gal4 as well as color depth MIP scores (Otsuna et al., 2018) from NeuronBridge (Clements et al., 2022) for neuron to neuron, neuron to Split-GAL4 and neuron to MultiColor FlpOut (MCFO) images of expression patterns. All scores are stored in the VFB Neo4j database. NBLAST scores are stored as a sparse matrix where scores below 0.25 are removed and the remaining scores are limited to the top 20 for any given neuron or expression pattern.

To test the efficacy of NBLAST and CDMIP similarity score queries in finding targeting split-GAL4 combinations for neuron types, we used associations between neuron types and split-Gal4 combinations curated from the literature and for which we have images (746 associations). We first found all individual neurons of each type for which a known targeting split Gal4 combination exists. For each type for which a known targeting split-Gal4 combination exists, we found the highest NBLAST or CDMIP score between individual neurons of this type and images of each Split-GAL4 combination in the database. To measure recall for a realistic browsing scenario, we tested whether known targeting split-GAL4 combinations were returned in the first 20 hits. We calculated precision across all returned results. This strategy prevents results from being overwhelmed by false positives in cases where there are many neurons or split-GAL4 combinations of a single type.

The VFB website is driven by a customized version of the Geppetto web framework (Cantarelli et al., 2018). The 2D slice viewer improves on the neuron/expression image overlap of the previous (Milyaev et al., 2012) VFB 1.0 viewer to allow multiple signal overlaps with true color blending. To achieve this, webGL 2D canvas color blending was used in combination with auto-assigned, maximally spread, LAB-space signal colors. This ensures the maximum possible color differentiation with a new feature allowing the user to select any point on the image showing all signals present at that point. This allows VFB to deliver a desktop stack scrolling experience by preemptively buffering neighboring slices for all displayed items and supporting mouse gestures for navigation through the stack.

### 2.4 Visualizing circuits and part-trees

A Neo4j query is used to automatically generate a browsable tree (template ROI browser, Figure 2C) for each template, based on the painted domains associated with it.

The circuit browser uses an implementation of Yen’s k-Shortest Path algorithm in the Neo4j Graph Data Science package v2.1 (https://neo4j.com/docs/graph-data-science/current/algorithms/yens) to find the k shortest, most highly weighted paths between two selected neurons in a connectome, filtering out connections with a weight below a specified threshold. The weight is stored as a Neo4j edge property and corresponds to the number of synaptic connections in a given direction between two neurons, where one presynaptic density to one T-bar corresponds to a single connection. Yen’s algorithm calculates the lowest weighted paths, so we need to invert the weights. In our current implementation we calculate inverted weight by subtracting from 5000, a weight just above the highest weighted synaptic connection (4299 connecting DPM_R (FlyEM-HB:5813105172) to APL_R (FlyEM-HB:425790257). We are likely to adopt a less arbitrary solution in future, but based on expert feedback, this tuning provides useful, intuitive results.

## 3 Results

### 3.1 Organization of data

Images on Virtual Fly Brain are cross registered to a growing set of standard 3D image templates covering all central nervous system regions and post-embryonic stages (Figure 1). Integrative templates for adult brain, adult ventral nerve cord and larval nervous system at instars 1 and 3, integrate cross-registered image data from many data sources. For example, our main adult brain template has almost 100,000 cross-registered images from 64 datasets, including connectomics data from electron and light microscopy images of neurons, lineage clones and expression patterns. These come from a mixture of small-lab datasets and large datasets imported *via* well-established pipelines from external databases including FlyCircuit (Chiang et al., 2010; Shih et al., 2015), Flylight (Meissner et al., 2022), CATMAID (Saalfeld et al., 2009) (multiple instances hosted on VFB servers) and neuPrint (Clements et al., 2020). Where they provide useful reference, VFB also includes templates for single datasets in their native space, including dense connectomes, standard parcellation references and images depicting the relationship of the CNS to musculature. In summary, this cross referencing of data at the image level underpins a central feature of VFB to support the integration and comparison of many disparate datasets from across the *Drosophila* community.

To support FAIR sharing, in particular, simple and consistent ways to access and reference data, every image on VFB is assigned a globally unique, persistent, resolvable identifier in the form of a URL. This is important not only for hosted data that has no associated identifiers, but also for the large numbers of images that are assigned local identifiers on other resources, as these local identifiers are not globally unique or easily resolvable without further information. For example, the multiple CATMAID instances hosted by VFB have clashing neuron IDs, so these IDs are not sufficient to resolve data on CATMAID without additional information about which CATMAID instance the neuron ID came from. In contrast, the VFB-assigned URL resolves to the relevant, persistent page on VFB, from which data can be downloaded and can also be used to access data *via* our API. Both site and API provide mappings to IDs and links to data on external sites.

### 3.2 Exploring neuroanatomy

The VFB web app consists of a set of widgets for exploring and displaying information about neuroanatomy, which can be arranged as desired, using an internal windowing system. Figure 1 shows the default layout and features content related to our running example—the neuron WEDPN2 (adult wedge projection neuron 2). A pair of image browsers displays the same content in 2D (Figure 1A) and 3D (Figure 1B), in this case images of a WEDPN2 neuron (green), the wedge brain region (pale blue/grey) and the expression pattern of a split-GAL4 combination that targets WEDPN2 (pink). A foldable parts tree (Figure 1C) can be used to select and color brain regions, in this case the wedge is selected and coloured. A layers tool (layers, Figure 1D) serves as both a key to displayed content, associating colors with image names and types, and a control panel for selecting, removing, hiding or recoloring content. Finally, a term Information window (Figure 1E) displays detailed information about selected content, as well as a set of queries allowing access to extended information. In this case, the selected content is a neuron type with symbol WEDPN2 (reflecting the typical way this neuron type is referred to), and a longer, more descriptive name that uniquely distinguishes it in the context of all *Drosophila* anatomy. Term information also includes, alternative names (synonyms), classification (e.g., WEDPN2 is classified as a wedge projection neuron, GABA-ergic neuron and a BAlp3 lineage neuron), relationships to other anatomical classes, a referenced description, examples images (2D projection), curated split-GAL4 drivers and queries.

All selected 3D images can be downloaded separately or in bulk, with downloads incorporating licensing and references, allowing users to use these in their own analysis in combination with local data.

Virtual Fly Brain makes it easy to find and integrate information about brain regions, neuron types and individual registered images *via* a range of different entry points: text search; point and click selection from images; queries for neurons by their location and properties; and data driven search.

#### 3.2.1 Text search

Users can search for neuroanatomical structures, driver expression patterns, cell-types or images starting from almost any name found in the literature using an intelligent, autocomplete-based search system (Figure 3) accessed from the header of all VFB pages (Figure 2, yellow arrowhead). Search works irrespective of the order of words used and covers curated synonyms as well as official names and symbols from DAO (Figure 3A). A set of search filters (Figure 3B) allows users to restrict search content positively or negatively by type (e.g., neuron, anatomy, expression pattern), stage (e.g. adult, larva) or data type (e.g. image).

#### 3.2.2 Point-and-click selection from images

Users can browse and select brain regions by pointing and clicking on the 2D slice browser or the tree browser, triggering display of reference information about the brain region and giving access to queries for neurons by location.

#### 3.2.3 Queries for neurons by their location and properties

VFB can also be used to explore neuroanatomy and find and select content *via* more sophisticated queries tailored to the content selected and driven by both data and information curated from the literature. For example, starting from a brain region, users can search innervating neuron types or images and can intersect these queries to refine them. Figure 4 shows an example of this type of query, finding images of wedge projection neurons that have some part in the lateral horn. Queries for neurons also include queries by lineage, e.g. WEDPN2 can be found from a query for components of ‘adult BAlp3 lineage clone'.

### 3.3 Data-driven search

In addition to semantic search, VFB features neuron structure searches that find images depicting neurons with similar location and morphology to that depicted in an input image. These searches are driven by NBLAST similarity scores (Bates et al., 2020b), precomputed by VFB, and color-depth Maximum Intensity Projection (color-depth MIP) scores (Otsuna et al., 2018), provided by NeuronBridge (Clements et al., 2022). The Janelia hemibrain (Scheffer et al., 2020) and FAFB (Zheng et al., 2018) and the many studies that have traced neuronal circuits in these, provide *de facto* reference image datasets for identifying neuron types using NBLAST.

Figure 5 shows an NBLAST search for potential types for an untyped neuron (Cha-F-600036) from the FlyCircuit dataset (Chiang et al., 2010). Multiple high scoring matches to typed neurons support the assignment of this neuron as a type of WEDPN2 neuron. Searches like this will become a critically important tool as we enter an era of comparative connectomics as resources to manually annotate new data cannot keep pace with high throughput data collection. For example, the FlyWire (Dorkenwald et al., 2020) project is generating a minimally annotated, dense reconstruction of the FAFB brain. Making sense of this data will require cross-sample mapping of neuron types *via* algorithms like NBLAST.

### 3.4 Finding transgenic driver lines

In order to genetically dissect neural circuit function, *Drosophila* neurobiologists need to precisely target specific types of neurons to experimentally manipulate their activity. This is typically achieved using a split-GAL4 driver system that initiates downstream expression at the intersection of two transgene expression patterns (Luan et al., 2006). In these types of experiments, the biggest bottleneck is finding combinations of driver lines that precisely target the neuron type of interest.

VFB features over 99,000 queryable records, curated from the literature, associating transgenes and split combinations, recorded using FlyBase Identifiers, with the anatomical structures and cell types in which they are expressed, curated using the DAO. This includes 1508 split-GAL4 combinations targeting almost 700 types of neuron. These are displayed in the Term Information window for each neuron type, for example, WEDPN2 is targeted by split-GAL4 combination “R66A08 ∩ R85A07” (Figure 2E). Novel combinations of hemidrivers can potentially be found from among the curated records linking full transgene expression patterns to neuron types. However, these results only scratch the surface of untested split-GAL4 driver combinations from among the millions that are possible.

VFB also features over 43,000 registered 3D images of transgene expression patterns covering 16,876 transgenic driver lines, including over 2700 covering 579 split-GAL4 combinations. As well as full expression pattern images, VFB also hosts almost 50,000 images of stochastically generated subsets of neurons from full GAL4 expression patterns and split-GAL4 combinations, generated by a variety of techniques, including Multi-Color Flip Out (MCFO) (Nern et al., 2015).

VFB can be used to query for potential split driver combinations targeting any neuron type for which an image is available, using NBLAST scores (Figures 6B–D) or color-depth maximum intensity projection (CDMIP) similarity scores (Otsuna et al., 2018) from NeuronBridge (Clements et al., 2022) (Figure 7). In the example shown in Figure 6C, one of the top three hits from an NBLAST search (R66A08 ∩ R85A07) is confirmed by information curated from the literature. Analysis of the ability of NBLAST queries from neurons to return associations between neuron types and split combinations curated from the literature, shows that 53% of curated matches are returned in the top 20 (aggregating individual images by neuron type and Split-Gal4 combination), with a precision of 26% (calculated using all returned results). The same analysis using CDMIP scores gives much lower recall (16%) in the top 20 hits and precision (19%), calculated using all returned results. Given the high false positive rate, results need to be screened by eye. Each potential driver line can be loaded onto the stack browser together with the query neuron to manually check the quality of the match (Figure 6D). Figure 7 shows CDMIP search returning images of subsets of neurons in MCFO images of full driver expression patterns, potentially finding new hemidriver combinations. Where multiple driver lines are identified that have little overlap, this can form the basis for an intersectional approach to target a neuron type more precisely.

### 3.5 Exploring connectomics

VFB includes connectomics data from multiple sources, encoded as directional pairwise links between individual neurons, with weight recorded as the number of synaptic connections. Where available, we also record directional pairwise links between neurons and the brain regions they innervate, again including weight as number of synapses. These data are used to generate direct reports of connectivity for specific neurons. The latter is also used to drive queries for neuron images by region (Figure 4).

Our circuit query tool allows users to find the shortest, most highly weighted paths between any two neurons in the same connectome (Figure 8). Users can specify a minimum weight for connections and the number of paths to return. For ease of viewing, the results are arranged in a graph with rows and columns, with the first and last columns being the start and end neurons specified in the query. Neurons in the circuit between these two, are arranged in order of the numbers of hops from the starting neuron. Higher ranking paths (by length and weight) are displayed in lower rows. Edges display weight (forward and reverse). Nodes (neurons) display type and gross classification (e.g. cholinergic, olfactory). All nodes are selectable, for display of term information, classification, images etc.

### 3.6 Exploring single cell transcriptomics

**FIGURE 9:**
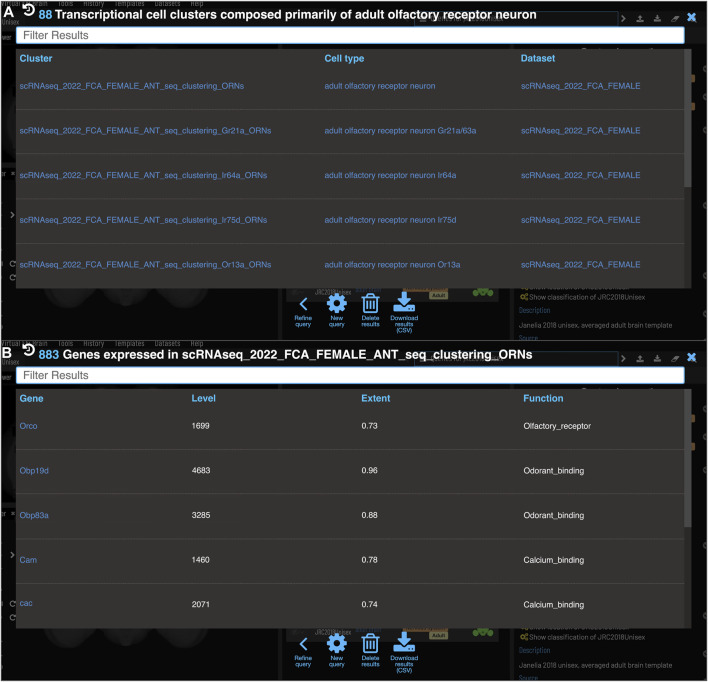
*Single Cell RNAseq* (not yet live) **(A)** Each transcriptional cluster is linked to a cell type in the *Drosophila* Anatomy Ontology (curation done by Single Cell Expression Atlas and FlyBase) facilitating searches based on cell type (typically more general types than we have for connectomics data). **(B)** Each gene expressed in more than half of the cells in a cluster will be viewable in VFB with its expression level and extent (proportion of cells in cluster that transcript was detected in) and semantic tags representing the gene’s function (based on GO and Gene Group annotations from FlyBase).

One of the major strengths of VFB’s semantic approach is the ease with which very different data types can be cross-integrated. VFB is working with FlyBase and the EBI single-cell atlas to annotate neuron types in single-cell transcriptomics data using the DAO. This allows queries for associated transcriptomics data from any cell type term or class in VFB. Figure 9A shows the results of a query for transcriptomics data for olfactory receptor neurons. The results table returns clusters, the datasets they are from and their cell type annotations. Figure 9B shows an example of summary expression data for one of these clusters, from the Fly Cell Atlas dataset (Li et al., 2022). For each gene these results show the level of expression, the proportion of expressing cells in the annotated cluster and semantic tags summarizing gene function, derived from Gene Ontology Molecular Function and Gene Group annotations in FlyBase (Figure 9B). Links from datasets to the EBI Single Cell Expression Atlas allow further exploration of data and download of cell-by-gene matrices and associated annotations for local analysis.

## 4 Discussion

### 4.1 Summary

VFB helps users build an integrated picture of the anatomical and molecular nature of neurons and the circuits they form by providing access to a wealth of curated information and data *via* multiple search and query systems and reports. A user might start with the name of a neuron type from the literature (Figure 3) and from there find a description, links to further papers (Figure 2), downloadable 3D images (Figures 2, 4), a list of known and potential driver combinations to use to target the neuron type (Figures 5–7) connectomics data (Figure 8), transcriptomics data (Figure 9). Or they might start from a phenotype caused by a particular split-GAL4 driver and from there, want to find neurons where this split-GAL4 driver is known, or predicted, to be expressed along with connectomics and transcriptomics reports for these neurons from multiple sources. They might be interested to find circuit paths between two neurons they believe to be targeted by two different split driver combinations that cause similar phenotypes when used to silence target neurons (Figure 8). In all cases, VFB supports rapid data discovery across datasets and provides a fast, accessible starting point for basic data exploration, while also supporting more advanced data exploration and analysis by providing data downloads and links to and identifiers for the same data in other tools and resources. Following FAIR data standards, all hosted data is downloadable under open licenses, with tracked provenance and rich metadata.

### 4.2 Relationship to other resources

Virtual Fly Brain adds unique value through comprehensive semantic and image-based data integration and inclusion of curated information from the literature. Related resources have some overlaps in functionality, but also have their own distinct functionalities and often include data that fall outside the current scope of VFB. VFB facilitates access to these resources *via* an extensive and flexible system of link-outs that link to the same data or entities on external sites. We are tightly integrated with FlyBase, which we link out to for all information on genetic features (genes, alleles, transgenes). We provide direct links from data on VFB to the same data on the sites of major data-providers (FlyCircuit (Chiang et al., 2010; Shih et al., 2015), FlyLight (Meissner et al., 2022), NeuronBridge (Clements et al., 2022), neuPrint (Clements et al., 2020) and CATMAID (Saalfeld et al., 2009)), which, while limited to their own data, each provide distinct query tools and additional meta-data over that captured by VFB. In the case of CATMAID, VFB is also the sole host for official, public facing CATMAID servers for multiple connectomics datasets, providing a vital service to the community by archiving these data sets in their original form as they are released, as well as integrating them into VFB.

VFB also provides a home for many datasets from independent labs that are not integrated by other resources and so would otherwise be inaccessible. This includes many independently generated Split-GAL4 datasets, registered image data for neuroblast lineage clones covering the adult brain, kindly contributed by the labs of Kei Ito and Tzumin Lee (Ito et al., 2013; Yu et al., 2013) and a dataset of 3D images of pharyngeal muscles, innervating motor neurons and split-GAL4 drivers (McKellar et al., 2020).

We also link out to related resources including InsectBrain DB (Heinze et al., 2021), which hosts 3D parcellation schemes and neuron images for brains of many insects and larvalbrain.org, which hosts reference information for larval anatomy and expression patterns. We currently do not link to Fruit Fly Brain Observatory/FlyBrainLab (Lazar et al., 2021), which is focussed on facilitating simulation, because their dynamically generated content pages do not easily support linking.

### 4.3 Future plans

VFB is built around a unique combination of curated knowledge and data, united by a common semantic schema: the same classifications and relationships are used to record the properties of types of neurons and individual instances of neurons; relationships between individuals can be associated directly with data, such as synapse number or NBLAST similarity score. Future extensions to VFB will continue to leverage this combination to provide unique functionality. For example, future versions of the connectomics browser will feature aggregation of synaptic strength to neuron types. We will continue to expand the inclusion of queryable data relevant to curated knowledge in VFB, including incorporating lineage inferred from primary neurite location and neurotransmitter predicted from the application of machine learning to electron microscopy data (Eckstein et al., 2020).

#### 4.3.1 Leveraging data to improve annotation; leveraging annotation to test algorithms

VFB increasingly combines curated knowledge claims with data relevant to those claims. For example, we include both curated claims about typing of individual neurons and the Split-Gal4 lines that target them as well as similarity scores that support these claims. This is potentially useful for finding mis-annotated data—e.g., if a neuron annotated as a specific cell type has a very low similarity score to all other neurons annotated to that type, the annotation is likely to be wrong and can be flagged as low reliability. In developing tools that use similarity scores, such as the proposed split finder service (described below), curated information can serve as a reference set to test and tune the tool, as demonstrated in the results described in Section 3.4.

#### 4.3.2 Split finder service

Currently, users wanting to find split-GAL4 drivers for neuron types on VFB can start from a neuron type and search for drivers curated as expressed in that neuron, based on the literature, or navigate down to an individual neuron to search by precomputed NBLAST or color-depth MIP scores. The latter functionality partially overlaps with that of NeuronBridge (Clements et al., 2022). We will extend NBLAST scores on VFB to include all MCFO images from FlyLight. Taking advantage of VFB semantics, we are working on a unified split-finder tool that supports a one click-search from neuron type for candidate split driver combinations and hemidrivers based on a combination of associations curated from the and similarity scores. Results will be viewable as color depth MIP images as these are faster to screen by eye for matches (Otsuna et al., 2018)**.**

#### 4.3.3 Supporting comparative connectomics

In the near future, VFB will ingest multiple large connectomics datasets with variable coverage and accuracy of neuron type annotation. BLAST-like algorithms, in the short-term NBLAST for morphology, but longer term supplemented by CBLAST (Scheffer et al., 2020) for connectivity and potentially methods that use subcellular features (Schubert et al., 2019; Zinchenko et al., 2022), will be critical to help users to interpret this data by facilitating prediction and assignment of neuron types. For example, a user finding paths between untyped neurons from FlyWire using our circuit browsing tool will be able to use NBLAST to find predicted types for neurons in the circuit, where these exist in other reference data sets. We will also investigate adding precomputed predicted neuron types based on NBLAST scores, with appropriate caveats, as a way of making browsing more efficient.

We are also about to release a service allowing registered neuron skeletons to be uploaded to the VFB site for viewing in the context of other 3D data and running NBLAST to predict neuron type.

#### 4.3.4 Integrating connectomics with transcriptomics

The ability to resolve neuronal cell types in *Drosophila* single cell RNAseq data to the same granularity as achieved when typing by morphology and connectomics is improving as larger numbers of cells are profiled (Bates et al., 2019) as developmental data is integrated, and with the help of bulk scRNAseq data for cells marked with Split-GAL4 drivers and mappings from these to cell types (Davis et al., 2020; Kurmangaliyev et al., 2020; Özel et al., 2021). The fruits of these approaches are most apparent in the optic lobe where we now have transcriptomics profiles of 200 cell types and the first integrated analysis across transcriptomic and connectomic data is now available (Kurmangaliyev et al., 2020; Özel et al., 2021).

While VFB currently only has limited scRNAseq data available (see Figure 9), the number of annotated datasets is growing rapidly thanks to a collaboration with FlyBase and the EBI single cell expression atlas. As the number of datasets and cell types covered by transcriptomics and connectomics data and mapped to split-GAL4 lines increases, mapping between datasets for combined analysis will become increasingly challenging. Providing uniform, standardized annotation of cell types and their classifications across all these data types and datasets puts VFB in a strong position to facilitate these combined analyses. The VFB web application provides mechanisms for browsing connections and finding paths in the connectomics data (Figure 8) and for rapidly navigating from this to transcriptomic profiles. More sophisticated analyses will be facilitated by accessing this data through the VFB_connect API.

#### 4.3.5 Improving 3D image visualizations

To limit load on user’s laptops, the 3D browser uses maximum projection point-cloud renderings of expression. While enabling multiple expression patterns to be overlaid, this approach is not ideal as it can throw away fine details and can fail to adequately reflect graded expression. We are working to transition the site to a full resolution display of graded expression data, taking advantage of advances in bandwidth and laptop GPUs.

#### 4.3.6 Adding anatomical context

VFB is in the process of ingesting multiple 3D datasets depicting the relationship of the nervous system to its inputs and outputs, including a complete 3D larva reconstructed from transmission electron microscopy data from serial sections and reconstruction of a fly leg, complete with muscles, sense organs and their innervating neurons from X-ray holographic nano-tomography data (Kuan et al., 2020).

#### 4.3.7 Improved links to the literature

While VFB already extensively links neuron types to relevant literature *via* curation, we are improving this using a natural language processing pipeline in order to provide, as far as possible, a complete and accurate coverage of literature links for all neuron types.

#### 4.3.8 User data upload

An interface allowing users to upload and annotate their own registered image data, receiving a globally unique, persistent, resolvable identifier in return, is currently in beta testing.

## 5 Conclusions

Virtual Fly Brain enables its users to search, browse, view, and download diverse, cross-integrated data relevant to developing and testing hypotheses about the circuit basis of complex behaviors in *Drosophila*. As the volume and diversity of both small and large *Drosophila* neurobiology datasets increases, and these are incorporated into VFB, the role of VFB as a data integrator will become increasingly important, especially for solving the problem of identifying neuron types in poorly annotated datasets and for finding reagents to target these neurons.

Similar data integration issues are faced in large atlasing projects in other species, including major planned atlases of mouse, human and non-human primates (Kaiser, 2022). The solutions developed by VFB are likely to prove useful in these cases too.

The semantic integration pipeline developed for VFB has already been re-used to underpin the Allen Brain Atlas cell type explorer (https://knowledge.brain-map.org/celltypes) a multi-modal single cell transcriptomics atlas of the mammalian primary motor cortex (Tan et al., 2021). It is also being re-used to drive autocomplete in the Cell Annotation Platform (http://celltype.info).

## Data Availability

Publicly available datasets were analyzed in this study. All data is available, following FAIR data sharing standards, through the VFB website (https://virtualflybrain.org) and API (https://pypi.org/project/vfb-connect/). The VFB site can be accessed through any computer + browswer with standard WebGL support, which can be tested at https://get.webgl.org/. The Drosophila Anatomy Ontology is available under a CC-BY-4.0 license from http://purl.obolibrary.org/obo/fbbt.owl. All code is available under open licenses at https://github.com/VirtualFlyBrain. Jupyter notebooks showing anaylsing the success of NBLAST and CDMIP scoresqueries in finding known split combinationsare available at https://github.com/VirtualFlyBrain/VFB_similarity_import/tree/vFrontiers/stats.
